# Posterior fossa choroidplexus papilloma in the pediatric population: case series and literature review

**DOI:** 10.1055/s-0043-1770351

**Published:** 2023-08-21

**Authors:** Rodrigo Inácio Pongeluppi, Matheus Fernando Manzolli Ballestero, Marcelo Volpon Santos, Ricardo Santos de Oliveira

**Affiliations:** 1 Universidade de São Paulo, Faculdade de Medicina de Ribeirão Preto, Hospital das Clínicas, Divisão de Neurocirurgia, Ribeirão Preto SP, Brazil.; 2 Universidade Federal de São Carlos, Departamento de Medicina, São Carlos SP, Brazil.

**Keywords:** Papilloma, Choroid Plexus, Pediatrics, Posterior Cranial Fossa, Papiloma do Plexo Coroide, Pediatria, Fossa Craniana Posterior

## Abstract

Choroid plexus papillomas (CPPs) are rare benign neoplasms which are particularly uncommon in the posterior fossa in children. We herein present a case series of five patients treated at a tertiary care hospital. A comprehensive literature review was also carried out. The patients treated at the tertiary care hospital were aged between 4 and 16 years. Gross total resection (GTR) was initially achieved in two patients. All patients showed clinical improvement. Moreover, 27 articles published between 1975 and 2021 were selected for the literature review, totaling 46 patients; with the 5 patients previously described, the total sample was composed of 51 cases, With a mean age was 8.2 years. The lesions were located either in the fourth ventricle (65.3%) or the cerebellopontine angle (34.7%). Hydrocephalus was present preoperatively in 66.7% of the patients, and a permanent shunt was required in 31.6% of the cases. The GTR procedure was feasible in 64.5%, and 93.8% showed clinical improvement. For CPPs, GTR is the gold standard treatment and should be attempted whenever feasible, especially because the role of the adjuvant treatment remains controversial. Neuromonitoring is a valuable tool to achieve maximal safe resection. Hydrocephalus is common and must be recognized and promptly treated. Most patients will need a permanent shunt. Though there is still controversy on its efficacy, endoscopic third ventriculostomy is a safe procedure, and was the authors' first choice to treat hydrocephalus.

## Resumo

Os papilomas do plexo coroide (PPCs) são neoplasias benignas raras e, na população pediátrica, são particularmente incomuns na fossa posterior. Apresentamos uma série de casos de cinco pacientes atendidos em um hospital terciário. Além disso, foi realizada uma ampla revisão da literatura. Os pacientes atendidos no hospital terciário tinham entre 4 e 16 anos. Ressecção macroscópica total (RMT) foi inicialmente realizada em dois pacientes. Todos os pacientes apresentaram melhora clínica. Além disso, 27 artigos publicados entre 1975 e 2021 foram selecionados para a revisão da literatura, totalizando 46 pacientes. Somados à série de casos atuais, encontramos 51 pacientes, com média de idade de 8,2 anos. As lesões localizavam-se no quarto ventrículo (65,3%) ou no ângulo pontocerebelar (34,7%). Hidrocefalia estava presente no pré-operatório em 66,7% dos pacientes, e derivação ventricular permanente foi necessária em 31,6% dos casos. A RMT foi possível em 64,5%, e 93,8% tiveram melhora clínica. Para os CPPs, a RMT é o tratamento padrão-ouro e deve ser tentado sempre que possível, especialmente porque ainda existem controvérsias quanto ao papel do tratamento adjuvante. A neuromonitorização é uma ferramenta importante para se atingir a máxima ressecção segura. A hidrocefalia é comumente vista nesses pacientes e deve ser identificada e tratada. A maioria dos pacientes irá precisar de uma derivação permanente. Apesar de persistirem controvérsias sobre sua eficácia, a terceiro-ventriculostomia endoscópica foi a primeira escolha para tratar a hidrocefalia na experiência dos autores e é uma opção segura.

## INTRODUCTION

Choroid plexus papillomas (CPPs) are rare neuroectodermal tumors that correspond to 0.4% to 0.6% of all neoplasms of the central nervous system. Its cause is still unknown; the role of some viruses in its pathogenesis has been hypothesized. 1

They are usually described as cauliflower-like neoplasms that are reddish and slow-growing. 2 The World Health Organization (WHO) classifies these tumors as CPP (grade 1) or atypical CPP (grade 2). 3 These tumors typically manifest with hydrocephalus, along with symptoms of raised intracranial pressure. 4 Surgical resection is the mainstay of treatment and adjuvant therapy is still controversial. 5

Most lesions are supratentorial and occur in the lateral ventricles, 6 7 usually in children within the first five years of life. 2 Posterior fossa lesions predominantly affect adults. 6

Hitherto, few cases of posterior fossa CCP have been described in the literature, and even fewer in the pediatric population. The most frequent clinical manifestations, optimal treatment, and clinical results are still poorly established for this population.

Thus, the aim of the present study is to describe the authors' experience with 5 cases of posterior fossa CPP in patients under 18 years of age. A comprehensive review of the current medical literature in English has also been carried out.

## METHODS

The present is a retrospective study of patients treated at a tertiary care hospital between 2007 and 2020. Data was extracted from the patients' electronic medical records and their confidentiality was preserved.

### Literature review

A literature review was undertaken to further characterize this rare pathology in the pediatric population. Thence, a MEDLINE/PubMed search was carried out in March 2021 using the following medical subject headings (MeSH) terms: *choroid plexus papilloma* and *cerebellum* or *posterior fossa* or *brainstem* or *cerebellopontine angle* .

We only included articles that described cases of grade-1 or grade-2 CPP in patients under 18 years of age at the time of diagnosis. Case series involving both adult and pediatric subjects, in which it was not possible to discriminate the age of the patients individually or to obtain the number of patients in the age group of interest, were excluded. The reference lists of the papers included were also searched to identify additional data sources.

## RESULTS

### Authors' experience

A total of 5 CPP patients were treated between 2007 and 2020 at our neurosurgical service, 2 male and 3 female subjects aged between 4 and 16 (mean: 8.2) years at the time of diagnosis. Four lesions were located in the fourth ventricle (FV) and one, in the left cerebellopontine angle (CPA).

The most common clinical symptoms were gait ataxia, headache, and seizures. Although epilepsy is not a common manifestation of posterior fossa lesions, 1 patient (#49) had a frontotemporal arachnoid cyst. This was most likely the cause of the epilepsy, which was posteriorly well controlled with medication alone. The other patient (#50) did not present any supratentorial lesions or other epileptic foci and improved after tumor resection. Moreover, he underwent video-electroencephalography (VEEG) monitoring, which did not demonstrate any focal epileptic activity.

At the initial presentation, the magnetic resonance imaging (MRI) scans of all patients showed lobulated lesions either in the FV (#47, #48, #49 and #50) or the CPA (#51), without any obvious infiltration of the adjacent cerebellum or brainstem. Marked contrast enhancement and variable “flow void” were found in all patients. Only 1 patient had gross calcifications (#48).

In total, 2 patients (#47 and #50) had preoperative hydrocephalus, which was treated with endoscopic third ventriculostomy (ETV). One of them (#50) showed clinical evidence of ETV failure secondary to postoperative ventriculitis and required ventriculoperitoneal shunting (VPS). The other patient (#47) had hydrocephalus in the postoperative period, requiring temporary external ventricular drainage, which, after 1 week, was successfully weaned off.

Suboccipital craniotomy and the telovelar approach were chosen for all FV lesions, except for patient #50, in whom the lesion was extruding through the cerebellar parenchyma. The cerebellopontine lesion of patient #51 was operated through a retromastoid craniotomy.

Gross total resection (GTR) was initially feasible in 2 patients (#49 and #51). In both cases, no infiltration to adjacent tissues was found. In patient #51, the tumor was closely attached to the brainstem; however, it did not preclude GTR. In another patient (#48), second-look surgery for a residual lesion was required and GTR was performed; the lesion was also described as encapsulated. Patients #47 and #50 presented with infiltrative/adhesive lesions and subtotal resection (STR) was performed. Interestingly, infiltrative and encapsulated lesions were found macroscopically regardless of the WHO grade. Neuromonitoring aided resection and intraoperative decisions in all cases.

Significant blood loss, requiring transfusion, was not observed. The surgical complications included transient neurological deficits, ventriculitis and cerebrospinal fluid (CSF) leaks. However, all of these complications were transient and managed accordingly, thus not affecting the overall clinical outcome.

In 2 cases (patients #47 and #48), histopathology confirmed atypical CPP (WHO grade 2), and 1 (patient #47) underwent postoperative radiotherapy (RT). In our institution, RT is usually prescribed for patients with WHO grade 2 and residual lesions.

The mean follow-up was of 86.8 (range: 12 to 151) months, and all patients showed clinical improvement. To date, no patients submitted to GTR without clear residual lesions on MRI presented tumor recurrence. These results are summarized, along with other literature data, in Table 1 .

**Table 1:** Detailed information on the cases reported in the literature and on the present case series

Author, year	Case	Age	Sex	Location	Symptoms	H/C	H/C treatment	Tumor treatment	Outcome	Notes
Raimodi and Gutierrez, 1975 9	1	N/A	N/A	FV	N/A	N/A	N/A	N/A	N/A	3 histologically-proven cases were described, but no further details were provided
	2	N/A	N/A	FV	N/A	N/A	N/A	N/A	N/A	
	3	N/A	N/A	FV	N/A	N/A	N/A	N/A	N/A	
Hammock et al., 1976 10	4	8 yo	F	CPA	CNP, C/S	Postop	VPS	STR	Improv	
Laurence, 1979 11	5	18 mo	F	FV	N/A	Preop	N/A	N/A	N/A	
	6	7 yo	F	CPA	N/A	Preop	N/A	N/A	N/A	
	7	15 yo	F	FV	N/A	Preop	N/A	N/A	N/A	
Guidetti and Spallone, 1981 12	8	13 yo	M	FV	N/A	N/A	N/A	N/A	Improv	
	9	3 yo	F	FV	N/A	N/A	N/A	N/A	Improv	
	10	7 yo	F	FV	N/A	N/A	N/A	N/A	Improv	
Masuzawa et al., 1981 13	11	7 yo	N/A	FV	H/A, N/V, CNP	N/A	N/A	STR + RT	N/A	Spinal drop metastasis
Zhang, 1982 14	12	6 yo	N/A	N/A	N/A	N/A	N/A	N/A	N/A	No details
Piguet and de Tribolet, 1984 15	13	5 yo	M	CPA	H/A, N/V, CNP	N/A	N/A	GTR	Improv	SAH upon presentation
Maria et al., 1986 16	14	4 yo	M	FV	N/A	N/A	N/A	N/A	N/A	Full text unavailable
Lippa et al., 1989 17	15	1 day	F	FV	Tense fontanelle	Preop	VPS	STR	**Death**	Congenital lesion
Shin et al., 2001 18	16	14 yo	F	CPA	N/A	Preop	N/A	N/A	N/A	
Rostasy et al., 2003 19	17	18 mo	F	FV	N/V	Absent	Absent	STR	Improv	
Doglietto et al., 2005 20	18	16 yo	M	CPA	H/A, CNP	Absent	Absent	N/S	Improv	Bilateral lesion
Stafrace and Molloy, 2008 21	19	4 days	N/A	CPA	Increasing head circumference	Pop	N/A	N/A	N/A	
Larysz et al., 2010 22	20	N/A	N/A	N/A	N/A	N/A	N/A	N/A	N/A	No details
Ogiwara et al., 2012 24	21	6 yo	M	FV	H/A	Preop	EVD	STR	N/A	
	22	4 yo	F	FV	C/S, CNP	Preop	EVD	STR	N/A	
Crawford et al., 2013 23	23	23 mo	M	FV	N/V and sleepiness	Postop	VPS	GTR	N/A	
Xiao et al., 2013 25	24	10 yo	M	CPA	H/A, N/V	Absent	Absent	PR	Improv	
Koh et al., 2014 26	25	N/A	N/A	FV	N/A	N/A	N/A	N/A	N/A	4 histologically-proven cases were described, but no further details were provided
	26	N/A	N/A	FV	N/A	N/A	N/A	N/A	N/A	
	27	N/A	N/A	CPA	N/A	N/A	N/A	N/A	N/A	
	28	N/A	N/A	CPA	N/A	N/A	N/A	N/A	N/A	
Prasad et al., 2014 27	29	8 yo	M	CPA	C/S, CNP	Preop	N/A	GTR	N/A	Also described by Prasad and Mahapatra (2015) 28
Prasad and Mahapatra, 2015 28	30	5 yo	M	FV	H/A, C/S	Absent	Absent	STR	Improv	
Cai et al., 2015 29	31	7 yo	F	FV	H/A, N/V	N/A	N/A	GTR	Improv	
Luo et al., 2016 30	32	7 yo	M	CPA	Mental retardation, C/S	Preop	N/A	GTR	N/A	
	33	14 yo	F	CPA	C/S, N/V	Preop	VPS	GTR	N/A	
Morshed et al., 2017 31	34	14 yo	M	FV	H/A, C/S, N/V	Preop	EVD	GTR	Improv	Spinal drop metastasis
Muñoz Montoya et al., 2019 32	35	13 yo	F	FV	H/A	Postop	VPS	N/A	Improv	
Trybula et al., 2020 33	36	24 mo	M	FV	Incidental finding	Preop	EVD	GTR	Improv	
	37	17 yo	F	FV	H/A, C/S, dizziness	Preop	EVD	GTR	Improv	
	38	17 yo	F	FV	Incidental finding	Absent	Absent	GTR	Improv	
	39	23 mo	F	FV	C/S	Preop	EVD	GTR	Improv	
	40	5 yo	F	FV	C/S	Preop	EVD	GTR	Improv	
	41	17 yo	F	FV	Backache and blurred vision	Preop	EVD	STR	Improv	
	42	5 yo	F	FV	H/A, C/S, N/V	Preop	EVD + ETV	STR	Facial and ocular palsy	
	43	5 yo	M	CPA	Incidental finding	Absent	Absent	GTR	Improv	
Gaddi et al., 2020 34	44	11 yo	M	CPA	H/A, C/S	Preop	Absent	GTR	Improv	
Adib et al., 2021 2	45	17 yo	M	CPA	N/A	N/A	N/A	GTR	N/A	
	46	11 yo	M	CPA	N/A	N/A	N/A	GTR	N/A	
Present case series	47	16 yo	M	FV	Gait ataxia and C/S	Preop	ETV	STR + RT	Improv	Reoperation 4 years after the first surgery (residual lesion)
	48	7 yo	F	FV	Headache	Postop	EVD	**GTR in a second look surgery (STR in the first procedure)**	CSF fistula; overall improv	Reoperation 2 years after the first surgery (residual lesion)
	49	7 yo	M	FV	Seizures	None	Absent	GTR	Improv	Frontotemporal arachnoid cyst (probable cause of epilepsy)
	50	4 yo	F	FV	Seizures and gait ataxia	Preop	ETV + VPS	STR	Ventriculitis; overall improv	
	51	7 yo	F	CPA	Tremor in hands	None	Absent	GTR	Transient hoarseness	

Abbreviations: C/S, cerebellar signs; CNP, cranial nerve palsy; CPA, cerebellopontine angle; ETV, endoscopic third ventriculostomy; EVD, external ventricular drain; F, female; FV, fourth ventricle; GTR, gross total resection; H/A, headache; H/C, hydrocephalus; Improv, clinical improvement; M, male; mo, months old; N/A, not available; N/S, surgical treatment not specified; N/V, nausea and vomiting; Postop, postoperative; PR, partial resection; Preop, preoperative; RT, radiotherapy; SAH, subarachnoid hemorrhage; STR, subtotal resection; VPS, ventriculoperitoneal shunt; yo, years old.

### Illustrative case

**Figure 1:**
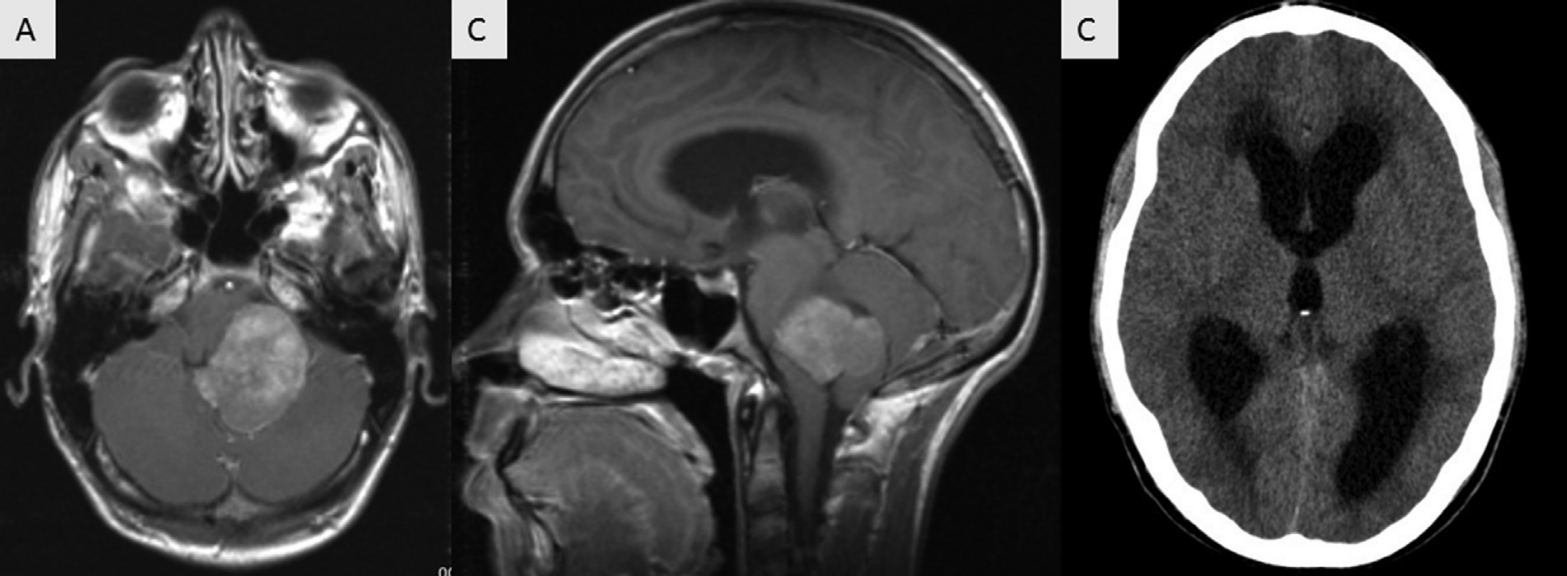
Preoperative magnetic resonance imaging (MRI) scans; axial ( **A** ) and sagittal images; ( **B** ) T1-weighted image (T1 WI) after contrast. Computed tomography (CT) scan showing supratentorial hydrocephalus ( **C** ).

A 16-year-old boy (patient #47) presented with a history of gait ataxia, dysmetria and dysdiadochokinesia in the previous month. Preoperative scans showed a heterogeneous FV lesion, contrast-enhancing, extending into the foramen of Luschka, and with supratentorial hydrocephalus ( Figure 1 ).

The patient underwent ETV and surgical resection of the lesion was performed five days later, via a suboccipital craniotomy and telovelar approach. Part of the tumor was infiltrating the brainstem and therefore was not removed, resulting in STR. This patient had no history of Li-Fraumeni syndrome, and since the residual lesion was small, conservative management was the initial choice of the multidisciplinary team.

**Figure 2:**
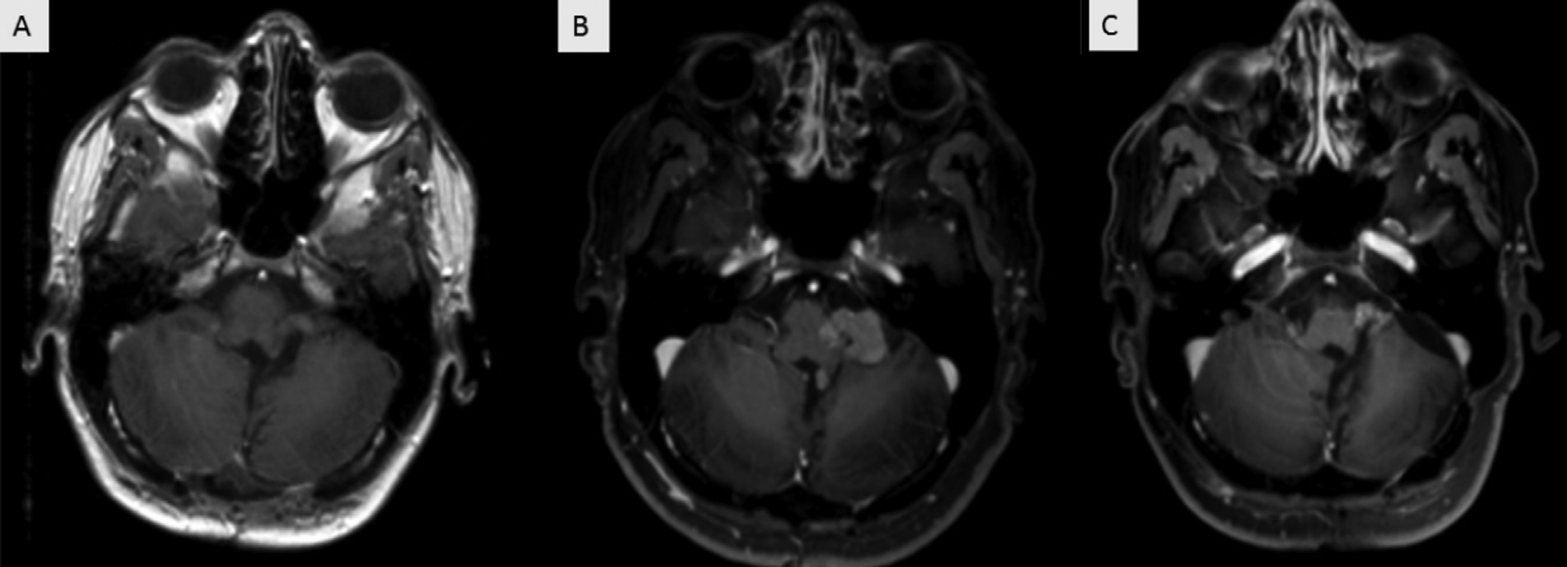
Postoperative T1 WI axial MRI scan one month ( **A** ) and four years ( **B** ) after the initial surgery. Tumor progression in the left cerebellopontine angle (CPA) is shown. Follow-up MRI showing a residual lesion in the left CPA ( **C** ).

However, this residual lesion grew progressively in the subsequent four years of follow-up, and reoperation was indicated. A retromastoid approach was then performed, and significant reduction of the residual tumor was achieved ( Figure 2 ).

Histopathology confirmed an atypical CPP (WHO grade 2). As previously mentioned, RT is indicated for residual lesions in this scenario. Thus, intensity modulated radiotherapy (iMRT) was performed postoperatively at a dose of 50.4 Gy. After 151 months of follow-up, the patient reported hearing loss on the left side, without any other neurological deficits, and a good cognitive outcome. The residual lesion has since remained stable ( Figure 2 ).

### Literature review

The literature review on MEDLINE/PubMed initially yielded 138 titles, 91 of which were actually on CPPs in children. This number was reduced to 20 after all abstracts were reviewed, but it increased again due to the addition of references not previously found. One of the published papers 8 was excluded because it was about one of the patients treated at our hospital and included in the present study. We ultimately selected 27 articles published between 1975 and 2021, comprising 46 single patients; including the ones herein reported, the total of patients was of 51.

The mean age of the patients was of 8.18 years, ranging from 4 days to 17 years; 32/49 (65.3%) lesions were located in the FV, and 17/49 (34.7%), in the CPA. The most frequent clinical manifestations were signs of cerebellar dysfunction (17/32), headache (13/32), cranial nerve palsy (7/32), and nausea or vomiting (7/32). Spinal drop metastases were documented in 2 patients (3.9%).

Hydrocephalus was present preoperatively in 22/33 (66.7%) patients, postoperatively in 3/33 (9.1%), and absent in 8/33 (24.2%) patients. Hydrocephalus requiring surgical treatment was observed in 18 cases. External ventricular drains (EVDs) were used in 10/19 (52.6%) patients, and permanent VPS was necessary in 6/19 (31.6%) patients. The ETV procedure was performed in 3/19 (15.8%) patients, 2 of them in the present series.

Among the patients whose surgical treatment was described, GTR was possible in 20/31 (64.5%), and RT was performed in 2 cases. In 27/51 cases, the clinical outcomes were reported; most of them (30/32) improved clinically, with only 1 death (1.9%). Moreover, in the medical literature, complications were only reported in 3 cases (5.8%): permanent cranial nerve palsy, ventriculitis, and transient hoarseness.

The results of the literature review are detailed in Table 1 , along with those of the present case series. 2 9 10 11 12 13 14 15 16 17 18 19 20 21 22 23 24 25 26 27 28 29 30 31 32 33 34

## DISCUSSION

Choroid plexus papillomas are extraordinarily uncommon in the posterior fossa. 28 Similar to previous reports, 4 headache, cerebellar signs, and cranial nerve palsy were the most common clinical manifestations in the pediatric population, as found in 53.1%, 40.6%, and 21.8% of our total sample respectively.

Notably, in the case series, 2 patients (40%) presented preoperatively with seizures, only 1 patient (20%), with headache, and no patients, with cranial nerve palsy. It is also noteworthy that in 1 case (20%) epilepsy was attributable to another intracranial lesion, reinforcing the fact that seizure is an uncommon presentation of posterior fossa lesions.

Metastatic tumor implants have already been described for both grade-1 and grade-2 CPPs. Most cases occurred in lesions of the posterior fossa and in adults. In the pediatric population, only 2 cases (3.9%) have been reported, both due to FV lesions. 2 31

The general appearance of CPPs on MRI scans is usually that of papillary or lobulated lesions with clear boundaries and moderate or strong gadolinium enhancement. Grade-1 and grade-2 CPPs cannot be distinguished based on the signal characteristics and enhancement patterns. 35 Peritumoral signal voids and calcifications have also been described. 18 All these typical radiological findings were observed in the present case series, albeit gross calcifications were found in only 1 (20%) patient.

Hydrocephalus is one of the hallmarks of choroid plexus tumors. Most patients present hydrocephalus at the time of diagnosis, 24 and that was also observed in the case series described in the present study. Some authors 5 have described an increase of 100% to 150% in the daily production of CSF; however, other mechanisms, such as obstruction of CSF pathways and arachnoid granulations, may justify shunt dependence in certain cases, even after GTR.

For obstructive hydrocephalus, ETV is known to yield superior results as compared to VPS, with equivalent successful outcomes and lower morbidity and mortality rates. 36 37 In pediatric posterior fossa lesions, higher failure rates are found in the first six months after ETV, but the complications of VPS outweigh those of ETV in the long term. 38 39 Currently, there are no specific recommendations for CPP patients, as it is a rare disease. Nonetheless, in the authors' opinion, as in the case of other posterior fossa tumors, ETV should be attempted, whenever feasible, to reduce VPS-related complications. In the literature, however, ETV has rarely been performed. We hypothesized that this may be also due to the low availability of ventricular endoscopy, especially for cases treated many years ago.

The need for permanent shunting after surgery ranges from 27.5 to 90%. 24 Moreover, children with FV lesions are more likely to require permanent VPS. 40 Overall, in the pediatric population, we found a lower rate than previously described, of 47.4%. However, it is worth mentioning that in 15 studies, which comprised 62.7% of patients, there was no information available regarding hydrocephalus treatment.

The gold standard treatment is GTR, which should be attempted whenever feasible. In a study published in 2002, 41 the overall 10-year survival rate was of 85% for GTR patients and of 56% for patients with partial resections. However, GTR was only feasible in 64.8% of all described cases. In the present case series, only 1 (20%) patient remained with a residual lesion; therefore, we endorse attempting maximal safe resection. Cranial nerve and brainstem adhesion may influence the decision to remove all of the visible tumor, as its manipulation may result in permanent deficits. In the authors' experience, neuromonitoring is very helpful for intraoperative decision making and to reduce complications.

The most widely used surgical routes in FV lesions are the transvermian and telovelar approaches; 40 the complication rates are similar for both, 42 but the latter is the choice of the authors of the present study. Exposure of the FV in the telovelar approach is satisfactory in most patients, and its floor can often be visualized early and protected. Deep rostral tumor attachment is the main limitation to this approach. 43

Benign CPP recurrence is even rarer, but this risk is estimated to be significantly higher in atypical CPP. 44 Yet, in the present series, no recurrence was observed in patients who underwent GTR. Similarly, other authors 24 found no recurrence in pediatric patients after a median follow-up of 78 months.

There is still controversy about the role of the adjuvant treatment. Though some authors 2 recommend adjuvant RT in cases of atypia or incomplete resection, others 45 46 advocate RT only for recurrent atypical lesions. Even when this treatment modality is indicated, the timing, dose, and extent of the field remains to be determined. 47 Radiosurgery has also been described as an option for small deep-seated lesions. 48 Chemotherapy has a limited role. Bevacizumab can be an option for disseminated and progressive disease. 49 In total, 1 (20%) patient in the current series and 1 case in the literature, both grade 2, underwent postoperative RT, with clinical improvement. Chemotherapy and radiosurgery have not been employed in pediatric CPP patients.

The clinical outcomes reported in the literature showed improvement in 93.8% of the patients. However, in 14 articles, comprising 47.1% of the patients, there was no description of the postoperative status, and good results may have been overestimated due to publication bias.

The overall complication rates for posterior fossa surgery has been reported 50 to be of 31.1%. This risk can be higher in children, especially in those with oncologic comorbidities, and infection rates of up to 25% have been reported. 51 We only observed 1 case (1.9%) of postoperative infection. Another frequent complication was CSF leak, observed in 7.1% of the patients 52 53 (1 case in the present series). Cerebellar mutism has been reported 52 in almost 30% of posterior fossa craniotomies in children, but it was not observed in the reported CPP cases. Permanent cranial nerve palsy has rarely been reported and was found in only 2 patients (3.9%) in the literature. Only 1 patient (1.9%) died in the sample herein analyzed.

In conclusion, posterior fossa CPPs are very rare in children. The present article aimed at reporting the relative heterogeneity of data availability and the diversity in the management of hydrocephalus, along with indications for adjuvant therapy and overall outcomes.

As expected in rare pathologies, clinical decisions must be individualized and/or extrapolated from the knowledge about similar diseases that are more common. Hydrocephalus must be recognized and promptly treated. The primary choice of most pediatric neurosurgeons is ETV, which should be attempted whenever feasible.

Surgical resection certainly remains the best therapeutic option for these neoplasms, and the goal should always be GTR, but surgical nuances, such as brainstem and fourth ventricle adhesion/infiltration, have to be appreciated. In our experience, neuromonitoring surely helps achieve maximal safe resection.

We can also conclude that the medical literature on this subject is still scarce, making individual experience very important when dealing with such pathologies. We believe that our experience together with the literature review herein presented can contribute to the decision-making process of clinicians who deal with this pathology.
